# Comparative Efficacy of 21 Treatment Strategies for Resectable Pancreatic Cancer: A Network Meta-Analysis

**DOI:** 10.3390/cancers16183203

**Published:** 2024-09-20

**Authors:** Fausto Petrelli, Roberto Rosenfeld, Antonio Ghidini, Andrea Celotti, Lorenzo Dottorini, Matteo Viti, Gianluca Baiocchi, Ornella Garrone, Gianluca Tomasello, Michele Ghidini

**Affiliations:** 1 Oncology Unit, Oncology Department, ASST Bergamo Ovest, Piazzale Ospedale 1, 24047 Treviglio, Italy; lorenzo_dottorini@asst-bgovest.it; 2 Oncology Unit, Fondazione IRCCS Ca’ Granda Ospedale Maggiore Policlinico, 20122 Milano, Italy; roberto.rosenfeld@policlinico.mi.it (R.R.); ornella.garrone@policlinico.mi.it (O.G.); michele.ghidini@policlinico.mi.it (M.G.); 3 Oncology Unit, Casa di cura Igea, 20129 Milano, Italy; a.ghidini@casadicuraigea.it; 4 Surgery Unit, ASST Cremona, 26100 Cremona, Italy; andrea.celotti@asst-cremona.it (A.C.); gianluca.baiocchi@unibs.it (G.B.); 5 Surgery Unit, ASST Bergamo Ovest, 24047 Treviglio, Italy; matteo_viti@asst-bgovest.it; 6 Oncology Unit, ASST Crema, 26013 Crema, Italy; gianluca.tomasello@asst-crema.it

**Keywords:** pancreatic cancer, operable, surgery, adjuvant, neoadjuvant, network meta-analysis

## Abstract

**Simple Summary:**

The primary treatment for operable pancreatic cancer involves surgery followed by adjuvant therapy, with an increasing use of perioperative or neoadjuvant chemotherapy. We performed a network meta-analysis to assess the comparative efficacy of various treatment approaches for resectable pancreatic cancer. A total of 24 studies were incorporated, comparing 21 treatments with surgery in isolation. Eleven treatments showed superior efficacy compared to surgery alone. Excluding Asian studies, the perioperative regimen of gemcitabine combined with nab-paclitaxel was the most effective regimen. The findings endorse the utilization of perioperative and multi-agent chemotherapy as the favored intervention for operable PC in Western nations.

**Abstract:**

The primary treatment for operable pancreatic cancer (PC) involves surgery followed by adjuvant therapy. Nevertheless, perioperative or neoadjuvant chemotherapy (CT) may be used to mitigate the likelihood of recurrence and mortality. This network meta-analysis (NMA) assesses the comparative efficacy of various treatment approaches for resectable PC. A thorough search was carried out on January 31, 2023, encompassing PubMed/MEDLINE, Cochrane Library, and Embase databases. We incorporated randomized clinical trials (RCTs) that compared surgical interventions with or without (neo)adjuvant or perioperative therapies for operable PC. We conducted a fixed-effects Bayesian NMA. We presented the effect sizes in terms of hazard ratios (HRs) for overall survival (OS) along with 95% credible intervals (95% CrIs). The treatment was deemed statistically superior when the 95% credible interval (CrI) did not encompass a null value (hazard ratio < 1). Treatment rankings were established based on the surface under the cumulative ranking curve (SUCRA). A total of 24 studies were incorporated, comparing 21 treatments with surgery in isolation. Eleven treatments showed superior efficacy compared to surgery alone, with HRs ranging from 0.38 for perioperative treatments to 0.73 for adjuvant 5-fluorouracil. After the exclusion of studies conducted in Asia, it was found that the perioperative regimen of gemcitabine combined with nab-paclitaxel was the most effective regimen (SUCRA, *p* = 0.99). The findings endorse the utilization of perioperative CT, especially multi-agent CT, as the favored intervention for operable PC in Western nations.

## 1. Introduction

In the last two decades, there has been a steady increase of 1% in the incidence of pancreatic cancer (PC) in both sexes. This places PC as the tenth most common cancer and the third leading cause of cancer-related deaths in 2024, following lung and colorectal cancers. Numerous studies conducted during this period have consistently reported poor survival outcomes for PC patients, with a marginal increase in the 5-year survival rate from 5% to 10% between 2000 and 2020. Notably, the highest survival rates are associated with early-stage radical surgical procedures combined with adjunctive oncological therapies [1,2,3,4].

Significant advancements in adjunctive treatment have been highlighted by the ESPAC-4 trial, which demonstrated a 7.5-month increase in overall survival (OS) with the addition of capecitabine (CAPE) to gemcitabine (GEM) therapy. Additionally, the PRODIGE-24 trial reported a 15.4-month extension in OS with the use of modified FOLFIRINOX (5-fluorouracil, irinotecan, and oxaliplatin triplet) compared to GEM alone [5,6]. Despite these advances, improving survival rates remains challenging, leading to the exploration of alternative therapeutic strategies.

Recent trends toward early intervention have prompted investigations into neoadjuvant treatments for resectable and borderline-resectable PC [7,8]. A meta-analysis focusing on mFOLFIRINOX in locally advanced PC showed promising outcomes, including a 24.2-month OS and significant tumor reduction, with resection rates of 25.9% and a remarkable 78.4% rate of radical (R0) resections in selected cohorts [9]. These outcomes appear to surpass those observed in the adjunctive setting, although a direct comparison is lacking. However, randomized clinical trials (RCTs) with a neoadjuvant focus on resectable or borderline-resectable PCs have produced inconclusive results [7,8,10]. The ESPAC-5 trial suggested a general benefit of neoadjuvant treatments over surgery alone but failed to establish a clear superiority between the CAPE-GEM and FOLFIRINOX regimens. Similarly, the Norpact-1 trial could not conclusively determine the efficacy of mFOLFIRINOX in a neoadjuvant setting. The lack of clarity in this matter has raised doubts about the applicability of advancements in treating locally advanced diseases to neoadjuvant therapies for early-stage PC [8,10,11].

Advancements in locoregional interventions, such as localized drug delivery systems, have garnered interest as alternatives to systemic therapy for pancreatic cancer. These therapies aim to minimize the side effects on healthy tissues and offer better control of localized tumors, addressing the limitations of systemic approaches. Additionally, novel systemic therapies, including more aggressive chemotherapy regimens and immunotherapy, are showing promise for PC. However, challenges such as the immunosuppressive nature of the tumor microenvironment persist, and ongoing research is investigating combined approaches to overcome these obstacles [12,13].

Nevertheless, radical surgery with or without adjunctive or neoadjuvant chemotherapy remains the foundation of treatment. Furthermore, comparisons between perioperative strategies for resectable tumors, such as neoadjuvant and adjunctive treatments, have not demonstrated significant differences in terms of R0 resection rates or survival outcomes [7,14]. Chemoradiotherapy (CTRT) has also not provided definitive evidence of its efficacy due to limited RCTs and available data. Although ESPAC-1 indicated the inferiority of CTRT, it is important to note that the regimen used was not aligned with current standards. Despite advancements in radiotherapy protocols, the optimal chemotherapeutic combination remains unconfirmed [14,15,16,17]. Consequently, the most effective perioperative treatment strategy for resectable PC, whether it involves CTRT, adjuvant chemotherapy (CT), or a combination thereof, remains unclear. The unresolved questions regarding treatment protocols, timing, and the potential for multimodal therapies are crucial. Thus, we present the first network meta-analysis (NMA) that compares randomized trials in resectable PC settings to determine the optimal strategy for maximizing OS benefit.

## 2. Materials and Methods

This study was conducted according to the established guidelines outlined in the PRISMA (Preferred Reporting Items for Systematic Reviews and Meta-Analyses) 2020 and the standards set forth in the PRISMA NMA statement. 

## 3. Search Strategy and Inclusion Criteria 

The search strategy and selection criteria involved the use of terms such as “pancreatic” or “pancreas” combined with “carcinoma”, “cancer”, or “adenocarcinoma”, as well as the inclusion of terms like “adjuvant”, “neoadjuvant”, “operable”, “resected”, “resectable”, and “randomized”. We systematically searched databases such as PubMed, Embase, and the Cochrane Library’s Central Registry of Controlled Trials. Additionally, we examined the reference lists of relevant studies. No restrictions were placed on language or publication year. Two independent evaluators (FP and MG) reviewed the research findings and resolved any discrepancies through collaborative group discussions until a unanimous agreement was reached. The inclusion criteria for studies were as follows: (a) prospective phase II/III randomized controlled trials (RCTs); (b) involving patients with upfront resectable or resected conditions; (c) including at least 50 participants; (d) reporting outcomes based on intention-to-treat (ITT) analysis; and (e) involving any treatment, including CT or chemoradiotherapy, either alone or in combination, pre- or post-surgery. Studies that did not meet these criteria were excluded, as well as non-RCTs such as meta-analyses, letters, case series, and reviews, studies lacking clear baseline characteristics, neoadjuvant studies on borderline-resectable or locally advanced pancreatic cancer (PC), studies on patients with non-adenocarcinoma histology, and studies without available overall survival (OS) data.

## 4. Quality of Studies and Data Extraction

The quality of the studies was assessed by two independent researchers (FP and MG) using Cochrane risk-of-bias tools. The assessment considered various aspects, including sequence generation, allocation concealment, blinding, incomplete data, selective reporting, and other potential sources of bias. Randomized controlled trials were categorized as having low, high, or unclear risk of bias. Discrepancies were addressed through discussions and consultations. The primary outcome measure used in this study was OS, which was expressed as hazard ratios (HRs) with 95% confidence intervals (CIs). If HRs were not directly reported, they were estimated from aggregate statistics using the method outlined by Tierney et al. in 2007 [18]. Data were analyzed based on an intention-to-treat (ITT) approach. The extracted information included author/year, country, study type, patient count, treatment arms, median follow-up, pathological nodal positive stage, rate of R1 resection, and postoperative mortality.

## 5. Statistical Analysis 

Heterogeneity between studies was evaluated using Q tests and I2 statistics. Depending on the I2 value (50% cutoff), fixed- or random-effects models were selected. Network meta-analysis was performed within a Bayesian framework using the “gemtc” package in the R software (version 4.0.2). The study was evaluated through consistency, global inconsistency, and local inconsistency assessments. The assessment of global inconsistency relied on comparing the fit of consistency and inconsistency models using the deviance information criterion (DIC). Consistent DIC values across the various models suggested a high level of coherence. Local inconsistency was assessed by comparing direct and indirect evidence within the Bayesian framework using node-splitting analysis. A significance level of *p* < 0·05 indicated significant inconsistency. The network meta-analysis employed both fixed effects and consistency models. Non-informative priors and posterior distributions were obtained using four Markov chains, with 40,000 iterations following 110,000 burn-ins and a thinning interval of 18. Network meta-analysis findings were reported as HRs with 95% CIs for different outcomes. Additionally, a ranking probability curve was calculated for each treatment regimen, indicating its effectiveness. The surface under the cumulative ranking (SUCRA) line was used to provide a quantitative summary and graphical representation of the cumulative ranking for each treatment, with 100% SUCRA indicating the optimal treatment and 0% indicating the worst [19,20,21].

## 6. Risk of Bias and Assessment of Evidence

The evaluation of GRADE was performed based on various network meta-analysis parameters. The risk of bias parameter assessed the potential for systematic deviation from the truth in the included studies, which could affect the certainty of the findings. The inconsistency referred to the discordance observed between direct and indirect evidence in the network meta-analysis. Intransitivity evaluated the feasibility of randomizing all competing interventions in a systematic review; this allowed for a unified multi-arm randomized trial where all treatments could be concurrently assessed within the same study population while controlling for other relevant variables such as stage, race, or age. Indirectness considered the differences among the populations and treatments, the variations in the outcomes of the studies compared to the populations and treatments, and the differences in the outcomes aimed at by the network meta-analysis, including the use of indirect comparisons. The presence of publication bias addressed the potential bias in the publication of these papers. Imprecision was primarily evaluated by examining the 95% confidence intervals to determine if they encompassed clinically significant effect sizes. Furthermore, the study assessed the adequacy of the available information. As this study did not involve human subjects, ethical approval or consent for participation was deemed unnecessary.

## 7. Results

**Figure 1:**
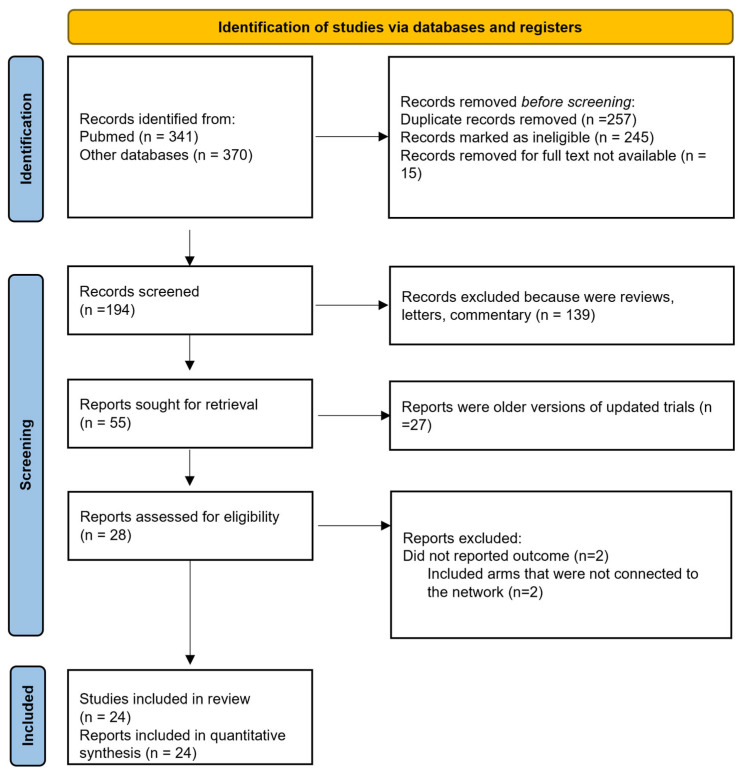
Flow diagram of included studies.

We identified 24 studies that were incorporated into our systematic review (Figure 1 and Table 1). All the studies included in the analysis were randomized, consisting of eight phase 2 trials, one phase 2–3 trial, and fifteen phase 3 trials. One study conducted by Reni et al. [22] employed a three-arm design that incorporated neoadjuvant, adjuvant, and perioperative strategies. Furthermore, three additional trials integrated a perioperative component. Notably, the study by Sohal et al. [14] included perioperative CT in both arms, whereas the studies by Sofferlein et al. and Labori et al. [10,23] included it only in the experimental arm. The study by Neoptolemos et al. [5] had a complex design, allowing clinicians to randomize patients into a two-by-two factorial design (observation, CTRT alone, CT alone, or both) or one of the main treatment comparisons (CTRT versus no CTRT or CT versus no CT), with only the latter two comparisons included in our primary analysis. Apart from the six Japanese trials, all studies involved patients from Western countries. Seven studies featured a CTRT arm, with two in the neoadjuvant setting and five in the adjuvant setting. Five studies incorporated arms that exclusively received surgical interventions. Nineteen publications examined the outcomes of surgical interventions followed by an experimental adjuvant CT arm, comparing it to surgery alone or surgery plus an adjuvant control arm. 

Four studies were excluded due to specific criteria: two did not report OS or survival curves, and two had treatment arms that were not linked within the network analysis.

The total patient population consisted of 6978 individuals, all diagnosed with resectable or resected PC in adjuvant studies.

**Figure 2:**
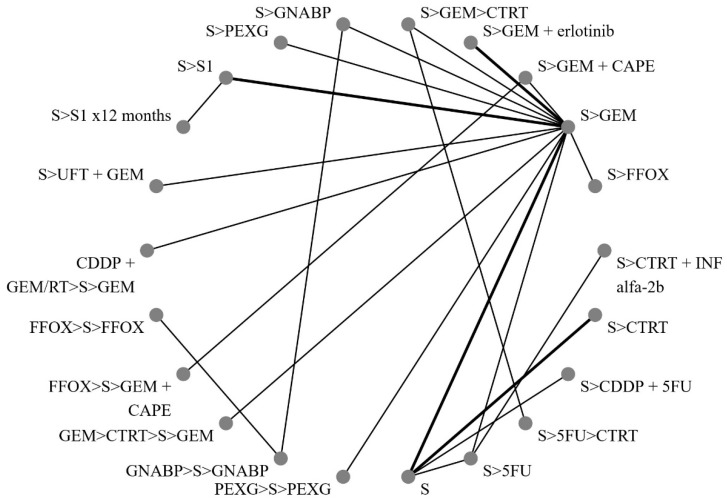
Network of direct treatment comparisons in operable pancreatic cancer. S, surgery; CT, chemotherapy; RT, radiotherapy; CDDP, cisplatin; GEM, gemcitabine; FFOX, FOLFIRINOX; CAPE, capecitabine; GNABP, gemcitabine + nab-paclitaxel; PEXG, cisplatin + epirubicin + capecitabine + gemcitabine; 5FU, 5-fluorouracil; IFN, interferon; ERLO, erlotinib; UFT, tegafur–uracil; >, followed by.

**Figure 3:**
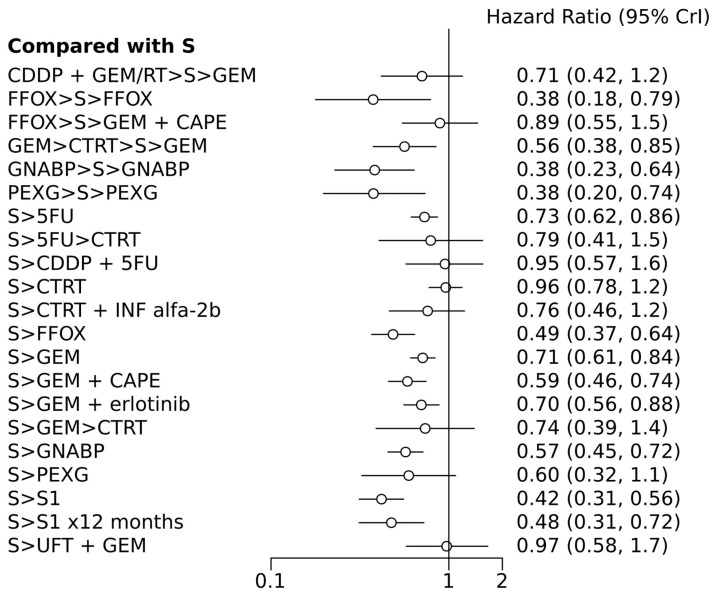
Forest plot of various treatment arms compared with surgery alone arm. S, surgery; CT, chemotherapy; RT, radiotherapy; CDDP, cisplatin; GEM, gemcitabine; FFOX, FOLFIRINOX; CAPE, capecitabine; GNABP, gemcitabine + nab-paclitaxel; PEXG, cisplatin + epirubicin + capecitabine + gemcitabine; 5FU, 5-fluorouracil; IFN, interferon; ERLO, erlotinib; UFT, tegafur–uracil; >, followed by.

Figure 2 illustrates the network of eligible comparisons for OS, while Figure 3 depicts the outcomes of the NMA for the primary endpoints comparing different regimens with surgery alone. Notably, perioperative FOLFIRINOX and GEM + nab-paclitaxel, adjuvant S1 for 12 months, CTRT followed by INF-alpha 2b, and 5FU followed by CTRT were not directly compared with the surgery-only arm.

Based on I2 < 50%, no significant heterogeneity was observed among the trials in the network; thus, a fixed-effects model was used. Fifteen trials had a low (*n* = 7), moderate (*n* = 1), and uncertain (*n* = 1) risk of bias. 

### 7.1. Overall Survival

In terms of efficacy, among the n =11 treatments, perioperative PEXG, FOLFIRINOX, GEM + nab-paclitaxel, neoadjuvant GEM followed by chemoradiotherapy (CTRT), adjuvant 5FU, adjuvant GEM, adjuvant FOLFIRINOX, adjuvant GEM + capecitabine (CAPE), adjuvant GEM + erlotinib, adjuvant GEM + nab-paclitaxel, and adjuvant S1 (for 6 or 12 months) were all superior to surgery alone, with HRs ranging from 0.38 for perioperative PEXG, FOLFIRINOX, and GEM + nab-paclitaxel to 0.73 for adjuvant 5FU (Table 2 and Table 3). Perioperative PEXG ranked first, with a *p*-value of 0.34. Adjuvant S1 demonstrated superior efficacy compared to various adjuvant GEM-based regimens, adjuvant 5-fluorouracil, adjuvant CTRT, or surgery alone. It is important to note that these findings were based on a study conducted exclusively within a Japanese population.

Adjuvant GEM + CAPE was more effective than adjuvant GEM, surgery alone, adjuvant CTRT, and neoadjuvant FOLFIRINOX, followed by surgery and other adjuvant CT. Adjuvant FOLFIRINOX demonstrated superiority over adjuvant GEM, adjuvant 5-fluorouracil, adjuvant 5-fluorouracil-based CTRT, adjuvant cisplatin + 5-fluorouracil, neoadjuvant FOLFIRINOX followed by surgery, other adjuvant CT, and surgery alone.

The SUCRA probabilities for the best treatment were 1 for adjuvant S1; 0.96 for perioperative PEXG; 0.95 for perioperative GEM + nab-paclitaxel; 0.95 for adjuvant S1 for 12 months; 0.92 for perioperative FOLFIRINOX and adjuvant GEM + nab-paclitaxel; 0.87 for adjuvant FOLFIRINOX and adjuvant GEM + CAPE; 0.88 for adjuvant GEM + nab-paclitaxel; and 0.69 for neoadjuvant GEM + CTRT. Other treatments were associated with lower SUCRA probabilities, as follows: neoadjuvant FOLFIRINOX followed by surgery and other adjuvant CT (0.09); surgery alone (0); adjuvant 5-fluorouracil (0.09); adjuvant 5-fluorouracil followed by CTRT (0.25); adjuvant cisplatin + 5-fluorouracil (0.07); adjuvant CTRT (0.003); adjuvant CTRT + interferon-alpha2b (INF-alfa2b) (0.27); adjuvant GEM (0.09); adjuvant GEM + erlotinib (0.12); adjuvant GEM followed by CTRT (0.36); and adjuvant tegafur–uracil (UFT) + GEM (0.05).

According to the random effects model, only four treatments have shown superiority over surgery alone: adjuvant FOLFIRINOX, S1, perioperative PEXG, and perioperative GEM + nab-paclitaxel. The DIC values were similar in the fixed- and random-effect models, with values of 49.3 and 49.9, respectively.

### 7.2. Sensitivity Analysis 

After excluding the phase 2 studies, 13 comparisons were included. Except for the CTRT-based regimens and adjuvant PEXG, all experimental treatments showed superiority compared to surgery alone, with HRs ranging from 0.38 to 0.73. Adjuvant S1 and perioperative PEXG have been identified as the two most effective treatments, with SUCRA probabilities of 0.99 and 0.96, respectively.

Six studies were solely dedicated to Asian patients. Upon excluding these studies, it was determined that ten different approaches demonstrated superiority over surgery alone, with HRs ranging from 0.37 to 0.72. The most effective regimen was perioperative GEM + nab-paclitaxel (administered for 2–3 cycles before and 3–4 cycles after surgery, with a P rank of 0.2 and a SUCRA probability of 0.99), followed by perioperative PEXG (administered for three cycles before and after surgery, with a P rank of 0.37 and a SUCRA probability of 0.96), and then perioperative FOLFIRINOX (administered for six cycles before and after surgery, with a P rank of 0.33 and a SUCRA probability of 0.93).

### 7.3. The Grading of the Evidence (Table 4)

Table 2 compares different treatments against each other in terms of overall survival, showing which are statistically superior based on HRs. Table 4 evaluates the quality of evidence for each treatment directly compared to surgery alone, providing an overall GRADE rating based on the level of bias, consistency, directness, and precision of the studies.

In line with the GRADE assessment, eight studies with a moderate-to-high risk of bias were included, resulting in a downgrade of the overall assessment. A total of 910 randomly selected patients self-identified as individuals of Asian descent. This suggests that race, in conjunction with imprecision (as indicated by the width of the confidence intervals), played a significant role in downgrading. A substantial level of evidence was demonstrated for perioperative GEM + nab-paclitaxel, PEXG, and FOLFIRINOX, as well as for adjuvant 5-FU, adjuvant GEM, adjuvant GEM + CAPE, adjuvant FOLFIRINOX, and adjuvant GEM + nab-paclitaxel compared to surgery alone. Furthermore, perioperative GEM + nab-paclitaxel and adjuvant FOLFIRINOX exhibited a higher level of evidence when compared to adjuvant CTRT.

**Table 4:** Grading of evidence of various treatment arms in comparison to surgery alone (according to GRADE classification).

Treatment 1	Treatment 2	Risk of Bias	Inconsistency	Indirectness	Publication Bias	Rating for NMA	Imprecision	Final Rating
**CDDP + GEM/RT > Surgery > GEM**	**Surgery**	Moderate	Not serious	Not serious	Not serious	Moderate	Serious	Very low
**FOLFIRINOX > Surgery>FOLFIRINOX**	**Surgery**	Low	Not serious	Not serious	Not serious	Moderate	Not serious (large effect)	**High**
**FOLFIRINOX > Surgery > adj CT**	**Surgery**	Low	Not serious	Not serious	Not serious	Moderate	Serious	Very low
**GEM>CTRT >Surgery > GEM**	**Surgery**	Low	Not serious	Not serious	Not serious	High	Not serious	**High**
**GEM + Nab-P > Surgery > GEM + Nab-P**	**Surgery**	Low	Not serious	Not serious	Not serious	High	Not serious	**High**
**PEXG > Surgery > PEXG**	**Surgery**	Low	Not serious	Not serious	Not serious	High	Not serious	**High**
**Surgery > 5FU**	**Surgery**	Low	Not serious	Not serious	Not serious	High	Not serious	**High**
**Surgery > 5FU > CTRT**	**Surgery**	Low	Not serious	Not serious	Not serious	Low	Serious	Very low
**Surgery > CDDP + 5FU**	**Surgery**	Moderate	Not serious	Serious	Serious	Moderate	Serious	Very low
**Surgery > CTRT**	**Surgery**	Moderate	Not serious	Serious	Serious	Moderate	Serious	Very low
**Surgery > CTRT + INF-alfa2b**	**Surgery**	Moderate	Not serious	Serious	Serious	Moderate	Serious	Very low
**Surgery > FOLFIRINOX**	**Surgery**	Low	Not serious	Not serious	Not serious	High	Not serious	**High**
**Surgery > GEM**	**Surgery**	Low	Not serious	Not serious	Not serious	High	Not serious	**High**
**Surgery > GEM + capecitabine**	**Surgery**	Low	Not serious	Not serious	Not serious	High	Not serious	**High**
**Surgery > GEM + erlotinib**	**Surgery**	Moderate	Not serious	Not serious	Not serious	Moderate	Not serious (large effect)	**Moderate**
**Surgery > GEM > CTRT**	**Surgery**	Low	Not serious	Not serious	Not serious	Low	Serious	Very low
**Surgery > GEM + Nab-P**	**Surgery**	Low	Not serious	Not serious	Not serious	High	Not serious	**High**
**Surgery > PEXG**	**Surgery**	Low	Not serious	Not serious	Not serious	High	Serious	**Moderate**
**Surgery > S1**	**Surgery**	Low	Not serious	Serious	Not serious	High	Not serious	**Moderate**
**Surgery > S1 × 12 months**	**Surgery**	Moderate	Not serious	Serious	Not serious	High	Not serious	**Moderate**
**Surgery + UFT + GEM**	**Surgery**	Moderate	Not serious	Serious	Not serious	Low	Serious	Very low

CT, chemotherapy; RT, radiotherapy; CDDP, cisplatin; GEM, gemcitabine; FOLFIRINOX, fluorouracil + irinotecan + oxaliplatin; Nab-P, nab-paclitaxel; PEXG, cisplatin + epirubicin + capecitabine + gemcitabine; 5FU, 5-fluorouracil; IFN, interferon; UFT, tegafur–uracil; >, followed by.

## 8. Discussion

The treatment of resectable PC has evolved significantly over the years, with the prevailing standard being upfront surgery followed by a six-month course of adjuvant CT, typically utilizing modified FOLFIRINOX (mFOLFIRINOX) for patients who are suitable candidates. This regimen has shown notable improvements in OS compared to other treatments. However, this standard approach is not without its challenges and limitations, prompting ongoing investigations into alternative strategies, such as neoadjuvant and perioperative therapies. The standard approach of upfront surgery followed by adjuvant CT with mFOLFIRINOX has demonstrated significant survival benefits. For example, the PRODIGE-24 trial highlighted a 15.4-month extension in OS with mFOLFIRINOX compared to gemcitabine alone. This regimen is particularly recommended for fit patients who can tolerate its intensity; however, for elderly or unfit patients, alternative treatments such as GEM and CAPE combination therapy or single-agent GEM are considered due to their better tolerability [23,24,25,26,27].

Despite these advancements, a substantial proportion of patients with resectable PC may not qualify for adjuvant CT due to poor postoperative performance status (PS). Studies have shown that only a fraction of patients who undergo primary resection are able to initiate or complete adjuvant CT. For instance, in the NEONAX trial, while 90% of patients completed preoperative GEM + nab-paclitaxel, only 58% of those who underwent primary resection could start adjuvant CT. This discrepancy underscores the challenges associated with postoperative recovery and the ability to sustain aggressive CT regimens post-surgery. Given the limitations of the standard adjuvant approach, there has been growing interest in neoadjuvant and perioperative therapies. Neoadjuvant therapy involves administering CT or chemoradiotherapy before surgery, with the aim of downstaging the tumor, increasing the likelihood of a successful resection, and addressing micrometastatic disease early in the treatment process. Perioperative therapy includes both pre- and post-surgery treatments, providing a comprehensive approach to managing the disease. The evidence supporting neoadjuvant therapy in resectable PC has been limited and conflicting. Various meta-analyses and randomized trials have yielded inconsistent results. For instance, the NORPACT-1 trial compared neoadjuvant FOLFIRINOX with upfront surgery and found no significant benefit in terms of resection outcomes. Additionally, different meta-analyses have reported varying conclusions regarding the efficacy of neoadjuvant therapy compared to upfront surgery followed by adjuvant CT [14,22,23,28,29]. Despite these mixed results, some studies have highlighted the potential benefits of neoadjuvant therapy. For example, FOLFIRINOX-based neoadjuvant therapy has shown promise in significantly downstaging borderline-resectable or locally advanced PC, with high rates of R0 resection, indicating the complete removal of the tumor with no microscopic residual disease. This suggests that neoadjuvant therapy could be particularly beneficial for patients with more advanced disease stages [30,31,32,33].

Our systematic review and NMA aimed to evaluate the comparative efficacy of various treatment approaches in the neoadjuvant and adjuvant settings for potentially resectable PC. The analysis excluded studies limited to Asian populations, particularly those involving adjuvant S1 due to its limited effectiveness outside Asia. The remaining studies identified perioperative regimens, particularly GEM + nab-paclitaxel, PEXG, and FOLFIRINOX as showing the best performance in terms of OS. The benefits of perioperative treatment strategies are multifaceted. Fractionating the treatment between pre- and postoperative phases allows for higher tumor shrinkage during the neoadjuvant phase and reduces the incidence of new intraoperative or postoperative metastases. Furthermore, perioperative treatments have not been associated with increased surgical complications and tend to decrease the frequency of treatment discontinuation due to toxicities or deteriorated PS. These advantages make perioperative regimens an attractive option for managing resectable PC. In the NEONAX trial, the completion rates for preoperative GEM + nab-paclitaxel were significantly higher compared to the initiation rates of adjuvant CT following primary resection. Similarly, the PACT 15 trial and studies by Sohal et al. [14] reported high completion rates for preoperative treatments compared to postoperative ones, underscoring the higher dose intensity and completion rates achievable with perioperative regimens.

Despite the promising results from perioperative therapies, several challenges and limitations must be considered. One major challenge is the clinical heterogeneity of the studies included in meta-analyses. The inclusion of studies with diverse patient populations, treatment protocols, and study designs introduces significant variability, potentially affecting the comparability and generalizability of the findings. Furthermore, meta-analyses are prone to publication bias, where studies with positive outcomes are more likely to be published, skewing overall findings toward more favorable results. Another limitation is the variability in dosages, timing, administration methods of treatments such as FOLFIRINOX, GEM + nab-paclitaxel, and adjuvant S1 among the included studies. This variation necessitates careful interpretation of meta-analysis outcomes and calls for standardized treatment protocols to ensure consistency and reliability. Additionally, the quality of the included randomized clinical trials significantly influences meta-analysis outcomes, with variations in study quality, such as blinding, allocation concealment, and loss to follow-up, potentially affecting the reliability of the findings. Finally, the small number of studies for certain treatments, such as targeted agents or special four-drug combinations (e.g., the Italian PEXG schedule), limits the ability to generalize the results widely.

Long-term survival, quality of life, and late treatment side effects are crucial factors influencing clinical decision-making in PC treatment. While many studies focus on median OS, long-term outcomes and patient quality of life are equally important. Treatments that extend life expectancy but significantly impair quality of life may not be preferable, emphasizing the need for balanced treatment strategies that consider both survival and patient well-being. Geographical and genetic factors also play a significant role in treatment efficacy, as evidenced by the preference for adjuvant S1 in Asian populations. This preference suggests that genetic differences might influence responsiveness to certain chemotherapeutic agents, highlighting the importance of personalized medicine tailored to genetic and regional differences [34,35].

The evolving landscape of PC treatment underscores the need for ongoing research to refine and optimize therapeutic strategies. Larger, well-designed randomized controlled trials are essential to establish definitive guidelines for the use of neoadjuvant and adjuvant therapies. Such trials should aim to address the limitations and variability identified in current studies, providing more robust evidence to guide clinical practice. Personalized approaches to treatment, considering patient and tumor characteristics, as well as regional and genetic differences, are crucial for maximizing therapeutic efficacy. The development of biomarkers to predict treatment response and tailor therapy accordingly could significantly enhance the outcomes for patients with resectable PC. Moreover, the role of immunotherapy in the neoadjuvant and adjuvant settings is an area of active investigation. Neoadjuvant therapy has been shown to reverse immunosuppression and deplete regulatory T cells and myeloid-derived suppressor cells, potentially leading to a better prognosis in locally advanced and borderline-resectable PC. The integration of immunotherapeutic agents with CT regimens could offer a synergistic approach to improving treatment outcomes [36,37,38,39].

The complexity and aggressive nature of PC necessitate a multifaceted approach to treatment, especially in resectable cases. While upfront surgery followed by adjuvant CT remains the standard, the exploration of neoadjuvant and perioperative therapies highlights the dynamic nature of current research and clinical practice. These alternative strategies aim to enhance the efficacy of treatment by addressing both the primary tumor and potential micrometastatic disease early on, thereby improving surgical outcomes and overall survival rates. One of the critical areas for future research is the identification and validation of biomarkers that can predict response to various CT regimens. Such biomarkers would enable a more personalized approach to treatment, ensuring that patients receive the most effective therapy based on their unique genetic and molecular profile. Additionally, ongoing trials investigating the combination of CT with novel immunotherapeutic agents hold promise for further improving outcomes in resectable PC. Moreover, the integration of advanced imaging techniques and minimally invasive surgical approaches could enhance the accuracy of tumor staging and resection, reducing postoperative complications and improving recovery times. The application of precision medicine principles, alongside advancements in surgical techniques and adjuvant therapies, will be pivotal in shaping the future of PC treatment.

Furthermore, implementing perioperative chemotherapy, such as FOLFIRINOX or gemcitabine-based regimens, requires substantial healthcare infrastructure. In low-resource settings, limited access to these drugs along with the high cost of supportive care and oncology specialists poses significant barriers. Strategies like prioritizing essential cancer medications and improving supply chain logistics can mitigate this. Also, some drugs may not be labeled for preoperative/early-stage indications. Administering perioperative chemotherapy requires trained healthcare professionals, including oncologists, nurses, and supportive care teams. Many regions face shortages of these skilled professionals, complicating the delivery of consistent perioperative care. Establishing training programs and international collaborations may help build local capacity. Effective perioperative chemotherapy requires consistent patient monitoring for treatment response and toxicity management. Limited access to regular follow-up care, imaging services, and laboratory tests can hinder optimal outcomes in some healthcare systems. Digital health tools, including telemedicine, can be employed to facilitate follow-up in resource-limited settings. Finally, socioeconomic disparities and cultural perceptions of cancer treatment may limit patient adherence to perioperative regimens. For example, patients may discontinue treatment due to transportation costs, fear of chemotherapy side effects, or a lack of trust in the healthcare system. Addressing these barriers requires culturally sensitive education and financial support programs.

The integration of multiomic data (radiomics, genomics, pathomics, and surgomics) with machine learning has the potential to revolutionize personalized treatment decisions for patients with resectable pancreatic cancer. When integrated with genomics and other omics data, radiomics can predict treatment outcomes by identifying specific imaging features associated with tumor biology and response to therapies. Whole-genomic sequencing identifies key genetic mutations and variations that drive cancer progression. Machine learning models can integrate this information with other omics data to predict personalized treatment regimens. Pathomics analyzes pathology data, such as histological images, to extract quantitative data that reflects the underlying biological processes of the tumor. Machine learning algorithms can integrate pathomic features with genetic and radiomic data to predict tumor behavior and tailor surgical approaches accordingly. Finally, surgomics involves data derived from surgical procedures, including the complexity of the operation and intraoperative variables. Combining surgomics with other omics data through machine learning can help predict surgical outcomes, recurrence rates, and the likelihood of complications. This integration allows for more informed decisions on whether to proceed with surgery or prioritize alternative treatments [40,41,42].

Despite the promising results, several challenges and limitations remain, including clinical heterogeneity, publication bias, and variability in treatment protocols. Ongoing research and larger randomized controlled trials are essential to establish definitive guidelines and optimize therapeutic strategies for resectable PC. Personalized approaches considering patient and tumor characteristics as well as regional and genetic differences are crucial for maximizing therapeutic efficacy and improving patient outcomes. The integration of targeted therapies with CT regimens represents a promising avenue for future research, potentially enhancing the efficacy of neoadjuvant and adjuvant treatments. As the landscape of PC treatment continues to evolve, ongoing research, personalized approaches, and the development of standardized protocols will be essential to improving outcomes and quality of life for patients with resectable PC.

In summary, while the paradigm of upfront surgery followed by adjuvant CT has significantly improved outcomes for patients with resectable PC, the exploration of neoadjuvant and perioperative therapies, personalized treatment strategies, and the integration of novel therapeutics promises to further enhance survival and quality of life for these patients. Ongoing research and innovation remain essential to achieving these goals. The treatment of resectable PC continues to evolve, with the prevailing standard being upfront surgery followed by adjuvant CT. However, the limitations associated with this approach, particularly for elderly or unfit patients, have prompted the exploration of neoadjuvant and perioperative therapies. Our systematic review and NMA support the use of multi-agent perioperative CT, particularly nab-paclitaxel + GEM, PEXG, and FOLFIRINOX, for fit patients. These regimens offer significant benefits in terms of overall survival and treatment completion rates compared to traditional adjuvant-only approaches.

## Figures and Tables

**Table 1:** Characteristics of included studies.

Author/Year	Type of Study	N° pts	Median Follow-Up (Months)	Exp Arm (n° Cycles)	Ctr arm (n° Cycles)	pN+ Stage (%) exp/ctr Arms	Pathological Poor Grade exp/ctr Arms	R1 (%) Exp/ctr Arms	Primary Endpoint	Resection Rates (%) exp/ctr Arms	Treatment Completion (%) exp/ctr Arms	Bias
**Abrams/2021**	Phase 2	163	42.5	Adj GEM + ERL (5 cycles)	Adj GEM (5 cycles)	74.8/72.4	-	16.4/16.6	OS	-	-	Moderate
**Conroy/2022**	Phase 3	493	69.7	Adj modified FOLFIRINOX (24 weeks)	Adj GEM (24 weeks)	57.2/74.5	-	64.6/80.3	DFS	-	-	Low
**Golcher/2015**	Phase 2	66	61	GEM/CDDP + RT → Surgery → Adj CT	Surgery → Adj CT	33/9	47.3/52.1	10.5/30.4	OS	57.5/69.7	21.2/30.3	Moderate
**Kayashima/2023**	Phase 2	170	-	Adj S1 (6 months)	Adj S1 (12 months)	54.9/56.1	-	9.8/8.5	2-year OS	-	44/64.7	Moderate
**Klinkenbijl/1999**	Phase 3	218	-	Adj FU + RT	Observation	35.5/40.7	27/18	19/24	OS	98.1/95.3	-	Uncertain
**Kosuge/2006**	Phase 2	89	44.8	Adj CDDP/FU (2 courses)	Surgery	26.6/20.4	-	0/0	OS	-	-	Moderate
**Labori/2024**	Phase 2	140	22.7	Neoadj FOLFIRINOX (2 months) → Adj CT	Surgery → Adj CT	71/86	-	44/61	18-month OS	82/89	66.2/74.6	Low
**Neoptolemos/2001**	Phase 3	285	10	Adj FUFA + RT	Adj FUFA	56/16	17/16	17/19	Death	-	-	Low
**Neoptolemos/2010**	Phase 3	1088	34.2	Adj GEM (6 months)	Adj FUFA (6 months)	73/70	24/25	35/35	OS	-	-	Low
**Neoptolemos/2017**	Phase 3	730	43.2	Adj GEM + CAPE (24 weeks)	Adj GEM (24 weeks)	79/82	41/39	61/60	OS	-	46/35	Low
**Oettle/2013**	Phase 3	354	136	Adj GEM (6 months)	Observation	71/73	35/38	19/15	DFS	-	-	Low
**Regine/2011**	Phase 3	451	17.7	Adj RT + GEM	Adj RT + 5FU	68/64	30/23	35/33	OS	-	-	Low
**Reni/2018**	Phase 2-3	88	55.4	Adj PEXG (6 cycles) Neoadj PEXG (3 cycles) → Surgery → Adj PEXG (3 cycles)	Adj GEM (6 months)	74/52/73	59/26/59	63/37/73	12-month EFS	90/90/84.6	70/86.9/41.1	Low
**Schmidt/2012**	Phase 3	132	42.7	Adj FU/CDDP/ IFNα-2b (5.5 weeks) + RT	Adj FUFA (6 months)	79/79	21/21	45/34	OS	-	-	Moderate
**Seufferlein/2022**	Phase 2	118	-	Periop nab-P/GEM (2 + cycles)	Adj GEM/nab-P (6 cycles)	33.9/37.3*	18.7/33.9	12.2/32.6	18-month DFS	69.5/78	42/25	Low
**Shimoda/2015**	Phase 2	57	-	Adj S1 (8 cycles)	Adj GEM (6 cycles)	-	-	6.9/12	DFS	-	37.9/42.8	High
**Sinn/2017**	Phase 3	436	54	Adj GEM + ERL (6 months)	Adj GEM (6 months)	64/66	33/34	-	DFS	-	-	Moderate
**Sohal/2021**	Phase 2	102	-	Periop mFOLFIRINOX (12 + 12 weeks)	Periop GEM/nab-P (9 + 9 doses)	60/55	-	15/15	2-year OS	72.7/70.2	49/40.4	Low
**Tempero/2023**	Phase 3	866	38.5	Adj nab-P/GEM (6 months)	Adj GEM (6 months)	72/72	23/26	24/23	DFS	-	66/71	Low
**Ueno/2009**	Phase 3	118	60.4	Adj GEM (3 months)	Surgery	67/70	9/7	19/13	OS	-	90/100	Low
**Uesaka/2016**	Phase 3	377	80.8	Adj S1 (4 cycles)	Adj GEM (6 cycles)	64/62	-	12/14	OS	-	28/42	Low
**Van Laethem/2010**	Phase 2	90	30.7	Adj GEM + RT	Adj GEM (4 cycles)	69/70	22/13	4/2	Feasibility and tolerability	-	78.5/95.2	Low
**Versteijne/2022**	Phase 3	248	59	GEM + RT → Surgery → Adj GEM (4 cycles)	Surgery → Adj CT (6 cycles)	35/82	31/29	28/57	OS	60/71.8	45.8/50.7	Low
**Yoshitomi/2008**	Phase 2	99	21	GEM + UFT (≥4 cycles)	GEM (≥4 cycles)	74/69.3	-	18/34.6	DFS	-	22/4.08	Moderate

**Table 2:** Comparison of the included interventions for OS–hazard ratio (95% CrI). Each cell gives the effect of the column-defining intervention relative to the row-defining intervention (yellow cells are those with significant results). The corresponding hazard ratios describe the effect size (if <1 treatment in column, better than the row schedule, and if >1 treatment in row, better than the corresponding column schedule). Level of evidence (L, M, H, or VL) of each direct or indirect comparison was graded according to GRADE method.

CDDP + GEM/RT>S>GEM	0.53 (0.22, 1.29)	1.25 (0.63, 2.49)	0.79 (0.42, 1.50)	0.54 (0.26, 1.10)	0.53 (0.23, 1.22)	1.41 (0.83, 2.40)	1.03 (0.61, 1.72)	1.12 (0.49, 2.54)	1.34 (0.64, 2.76)	1.35 (0.77, 2.39)	1.07 (0.53, 2.11)	0.68 (0.39, 1.19)	1 (0.61, 1.66)	0.82 (0.48, 1.41)	0.99 (0.58, 1.68)	1.04 (0.47, 2.3)	0.8 (0.47, 1.36)	0.84 (0.38, 1.8)	0.59 (0.34, 1.03)	0.67 (0.35, 1.26)	1.37 (0.67, 2.8)
	FFOX>S>FFOX	2.36 (1.01, 5.61) M	1.5 (0.66, 3.42)	1.01 (0.58, 1.75)	1. (0.37, 2.65)	2.66 (1.26, 5.61) H	1.94 (0.92, 4.03)	2.12 (0.79, 5.49)	2.52 (1.01, 6.18) M	2.55 (1.16, 5.53) M	2.01 (0.85, 4.73)	1.29 (0.6, 2.77)	1.89 (0.91, 3.9)	1.55 (0.73, 3.29)	1.86 (0.88, 3.94)	1.96 (0.75, 4.99)	1.51 (0.73, 3.06)	1.57 (0.62, 4.05)	1.11 (0.51, 2.38)	1.26 (0.55, 2.88)	2.59 (1.06, 6.34)
		FFOX>S>GEM + CAPE	0.63 (0.35, 1.15)	0.43 (0.21, 0.83) M	0.424 (0.194, 0.936) M	1.12 (0.68, 1.82)	0.81 (0.5, 1.32)	0.89 (0.4, 1.97)	1.06 (0.52, 2.13)	1.08 (0.63, 1.82)	0.85 (0.44, 1.64)	0.54 (0.32, 0.91) M	0.79 (0.5, 1.26)	0.65 (0.42, 0.99) M	0.78 (0.47, 1.27)	0.83 (0.38, 1.78)	0.64 (0.39, 1.04)	0.66 (0.31, 1.4)	0.47 (0.27, 0.78) M	0.53 (0.29, 0.97) M	1.09 (0.55, 2.17)
			GEM>CTRT>S>GEM	0.67 (0.36, 1.25)	0.66 (0.31, 1.41)	1.77 (1.18, 2.65) M	1.29 (0.86, 1.93)	1.4 (0.66, 3)	1.69 (0.87, 3.18)	1.7 (1.06, 2.7) L	1.34 (0.72, 2.45)	0.86 (0.55, 1.33)	1.26 (0.86, 1.84)	1.03 (0.68, 1.58)	1.24 (0.82, 1.87)	1.3 (0.64, 2.7)	1.01 (0.66, 1.53)	1.05 (0.52, 2.13)	0.74 (0.47, 1.15)	0.84 (0.49, 1.44)	1.73 (0.92, 3.22)
				GNABP>S>GNABP	0.98 (0.43, 2.22)	2.61 (1.56, 4.37) H	1.91 (1.14, 3.15) M	2.08 (0.91, 4.68)	2.47 (1.2, 5.02) M	2.5 (1.44, 4.35) H	1.98 (0.99, 3.89)	1.27 (0.74, 2.17)	1.86 (1.14, 3.03) M	1.52 (0.91, 2.57)	1.82 (1.09, 3.07) M	1.93 (0.87, 4.26)	1.48 (0.94, 2.35)	1.55 (0.72, 3.36)	1.09 (0.63, 1.87)	1.24 (0.66, 2.3)	2.55 (1.26, 5.17) L
					PEXG>S>PEXG	2.65 (1.35, 5.07) H	1.93 (0.99, 3.70)	2.09 (0.84, 5.24)	2.5 (1.1, 5.68) M	2.54 (1.26, 5.02) M	2.01 (0.89, 4.43)	1.28 (0.64, 2.5)	1.89 (0.98, 3.57)	1.54 (0.79, 2.99)	1.85 (0.95, 3.55)	1.95 (0.8, 4.76)	1.51 (0.77, 2.9)	1.57 (0.65, 3.72)	1.1 (0.55, 2.21)	1.26 (0.58, 2.66)	2.59 (1.15, 5.84) L
						S	0.73 (0.61, 0.86) H	0.79 (0.40, 1.54)	0.95 (0.57, 1.55)	0.96 (0.77, 1.18)	0.76 (0.46, 1.22)	0.48 (0.36, 0.64) H	0.71 (0.6, 0.83) H	0.58 (0.45, 0.74) H	0.7 (0.55, 0.88) M	0.73 (0.39, 1.38)	0.57 (0.45, 0.71) H	0.59 (0.32, 1.09)	0.41 (0.31, 0.55) M	0.47 (0.31, 0.72) M	0.97 (0.57, 1.65)
							S>5FU	1.08 (0.56, 2.10)	1.3 (0.76, 2.19)	1.31 (1., 1.73) L	1.04 (0.65, 1.62)	0.66 (0.5, 0.86) M	0.97 (0.85, 1.11)	0.8 (0.63, 1.01)	0.96 (0.77, 1.19)	1.01 (0.53, 1.88)	0.78 (0.63, 0.96) M	0.81 (0.44, 1.48)	0.57 (0.43, 0.75) M	0.65 (0.43, 0.98) L	1.33 (0.79, 2.25)
								S>5FU>CTRT	1.2 (0.52, 2.71)	1.21 (0.59, 2.43)	0.95 (0.42, 2.12)	0.61 (0.3, 1.21)	0.89 (0.47, 1.71)	0.73 (0.37, 1.44)	0.88 (0.45, 1.72)	0.93 (0.76, 1.13)	0.72 (0.36, 1.39)	0.75 (0.31, 1.8)	0.52 (0.26, 1.05)	0.6 (0.28, 1.26)	1.22 (0.54, 2.78)
									S>CDDP + 5FU	1.01 (0.59, 1.74)	0.79 (0.39, 1.6)	0.51 (0.28, 0.9) M	0.74 (0.44, 1.27)	0.61 (0.35, 1.075)	0.73 (0.43, 1.28)	0.77 (0.35, 1.73)	0.6 (0.34, 1.04)	0.62 (0.28, 1.36)	0.44 (0.24, 0.79) L	0.5 (0.26, 0.96) L	1.02 (0.49, 2.11)
										S>CTRT	0.79 (0.46, 1.33)	0.5 (0.35, 0.72) H	0.74 (0.57, 0.96) M	0.6 (0.43, 0.84) M	0.72 (0.53, 0.99) M	0.76 (0.39, 1.5)	0.59 (0.43, 0.81) M	0.61 (0.32, 1.18)	0.43 (0.3, 0.62) M	0.49 (0.31, 0.79) L	1.01 (0.57, 1.78)
											S>CTRT + INF alfa-2b	0.64 (0.37, 1.09)	0.93 (0.58, 1.52)	0.77 (0.46, 1.28)	0.92 (0.56, 1.54)	0.97 (0.45, 2.1)	0.75 (0.46, 1.24)	0.78 (0.37, 1.68)	0.55 (0.32, 0.93) L	0.62 (0.34, 1.15)	1.28 (0.64, 2.57)
												S>FFOX	1.46 (1.16, 1.85)	1.2 (0.89, 1.62)	1.44 (1.08, 1.92) L	1.51 (0.79, 2.92)	1.17 (0.88, 1.56)	1.22 (0.65, 2.32)	0.86 (0.61, 1.2)	0.97 (0.62, 1.55)	2 (1.15, 3.48)
													S>GEM	0.82 (0.67, 0.98) M	0.98 (0.83, 1.16)	1.03 (0.55, 1.9)	0.8 (0.68, 0.943 M	0.83 (0.46, 1.49)	0.58 (0.46, 0.74) M	0.66 (0.45, 0.99) VL	1.36 (0.83, 2.25)
														S>GEM + CAPE	1.19 (0.93, 1.53)	1.25 (0.66, 2.40)	0.97 (0.76, 1.25)	1.01 (0.55, 1.87)	0.71 (0.52, 0.97) L	0.81 (0.53, 1.25)	1.66 (0.98, 2.86)
															S>GEM + ERLO	1.05 (0.56, 1.98)	0.81 (0.64, 1.03)	0.85 (0.45, 1.55)	0.59 (0.44, 0.8) L	0.67 (0.44, 1.03)	1.38 (0.81, 2.36)
																S>GEM>CTRT	0.77 (0.4, 1.46)	0.8 (0.34, 1.89)	0.56 (0.29, 1.09)	0.64 (0.31, 1.32)	1.32 (0.59, 2.93)
																	S>GNABP	1.04 (0.56, 1.91)	0.73 (0.55, 0.98) VL	0.83 (0.55, 1.27)	1.7 (1, 2.88)
																		S>PEXG	0.7 (0.37, 1.31)	0.8 (0.39, 1.6)	1.64 (0.75, 3.56)
																			S>S1	1.13 (0.84, 1.54)	2.32 (1.34, 4.05)
																				S>S1 x12 months	2.04 (1.08, 3.83)
																					S>UFT + GEM

H, high level of evidence; M, moderate level of evidence; L, low level of evidence; S, surgery; CT, chemotherapy; RT, radiotherapy; CDDP, cisplatin; GEM, gemcitabine; FFOX, FOLFIRINOX; CAPE, capecitabine; GNABP, gemcitabine + nab-paclitaxel; PEXG, cisplatin + epirubicin + capecitabine + gemcitabine; 5FU, 5-fluorouracil; IFN, interferon; ERLO, erlotinib; UFT, tegafur–uracil; >, followed by.

**Table 3:** Rank probabilities table (rank 22 represents the worst treatment and rank 1 the best treatment, with corresponding probabilities).

	Rank 22	Rank 21	Rank 20	Rank 19	Rank 18	Rank 17	Rank 16	Rank 15	Rank 14	Rank 13	Rank 12	Rank 11	Rank 10	Rank 9	Rank 8	Rank 7	Rank 6	Rank 5	Rank 4	Rank 3	Rank 2	Rank 1
CDDP + GEM/RT > S > GEM	0.029	0.031	0.036	0.050	0.067	0.078	0.074	0.065	0.060	0.058	0.066	0.071	0.067	0.055	0.049	0.046	0.036	0.025	0.019	0.011	0.007	0.003
FFOX > S > FFOX	0.002	0.001	0.002	0.003	0.004	0.006	0.008	0.007	0.009	0.009	0.011	0.015	0.020	0.025	0.031	0.037	0.049	0.063	0.076	0.108	0.197	0.317
FFOX > S > GEM + CAPE	0.134	0.105	0.095	0.108	0.114	0.096	0.082	0.059	0.041	0.038	0.035	0.031	0.024	0.015	0.010	0.006	0.003	0.002	0.001	0.001	0.000	0.000
GEM > CTRT > S > GEM	0.001	0.001	0.002	0.004	0.011	0.017	0.020	0.026	0.030	0.039	0.057	0.074	0.099	0.104	0.107	0.108	0.099	0.080	0.059	0.035	0.020	0.008
GNABP > S > GNABP	0.000	0.000	0.000	0.000	0.000	0.000	0.001	0.001	0.002	0.003	0.004	0.006	0.010	0.016	0.022	0.034	0.054	0.080	0.108	0.189	0.300	0.168
PEXG > S > PEXG	0.000	0.001	0.001	0.001	0.004	0.003	0.005	0.006	0.005	0.007	0.010	0.013	0.020	0.025	0.029	0.039	0.055	0.070	0.080	0.146	0.136	0.344
S	0.087	0.239	0.278	0.214	0.112	0.053	0.015	0.003	0.001	0.000	0.000	0.000	0.000	0.000	0.000	0.000	0.000	0.000	0.000	0.000	0.000	0.000
S > 5FU	0.000	0.000	0.001	0.008	0.036	0.089	0.142	0.181	0.187	0.148	0.107	0.063	0.028	0.008	0.002	0.001	0.000	0.000	0.000	0.000	0.000	0.000
S > 5FU > CTRT	0.107	0.081	0.065	0.066	0.081	0.084	0.069	0.050	0.047	0.045	0.048	0.048	0.039	0.038	0.035	0.026	0.023	0.018	0.013	0.010	0.006	0.003
S > CDDP + 5FU	0.221	0.128	0.102	0.102	0.094	0.082	0.065	0.044	0.030	0.027	0.026	0.022	0.018	0.013	0.009	0.007	0.005	0.003	0.002	0.001	0.000	0.000
S > CTRT	0.093	0.154	0.190	0.184	0.157	0.102	0.061	0.033	0.014	0.005	0.003	0.002	0.001	0.000	0.000	0.000	0.000	0.000	0.000	0.000	0.000	0.000
S>CTRT + INF alfa-2b	0.040	0.044	0.051	0.070	0.087	0.097	0.087	0.075	0.063	0.054	0.060	0.061	0.052	0.045	0.034	0.029	0.021	0.013	0.009	0.005	0.002	0.001
S > FFOX	0.000	0.000	0.000	0.000	0.000	0.000	0.000	0.000	0.001	0.002	0.007	0.014	0.029	0.050	0.087	0.146	0.200	0.187	0.143	0.083	0.038	0.011
S > GEM	0.000	0.000	0.000	0.001	0.007	0.031	0.088	0.170	0.226	0.227	0.156	0.071	0.020	0.002	0.000	0.000	0.000	0.000	0.000	0.000	0.000	0.000
S > GEM + CAPE	0.000	0.000	0.000	0.000	0.000	0.001	0.003	0.008	0.013	0.035	0.070	0.117	0.167	0.186	0.168	0.123	0.065	0.029	0.010	0.003	0.001	0.000
S > GEM + ERLO	0.000	0.001	0.001	0.007	0.027	0.054	0.088	0.117	0.142	0.153	0.149	0.128	0.077	0.036	0.013	0.005	0.001	0.000	0.000	0.000	0.000	0.000
S > GEM > CTRT	0.016	0.073	0.057	0.053	0.065	0.081	0.085	0.065	0.050	0.050	0.055	0.062	0.054	0.048	0.044	0.039	0.032	0.026	0.020	0.014	0.011	0.004
S > GNABP	0.000	0.000	0.000	0.000	0.000	0.000	0.001	0.002	0.008	0.019	0.045	0.092	0.147	0.198	0.204	0.155	0.087	0.031	0.010	0.001	0.000	0.000
S > PEXG	0.015	0.014	0.018	0.022	0.034	0.042	0.044	0.042	0.038	0.044	0.052	0.063	0.069	0.069	0.066	0.071	0.070	0.064	0.057	0.046	0.038	0.026
S>S1	0.000	0.000	0.000	0.000	0.000	0.000	0.000	0.000	0.000	0.000	0.000	0.001	0.002	0.004	0.011	0.029	0.065	0.152	0.251	0.239	0.171	0.075
S > S1 × 12 months	0.000	0.000	0.000	0.000	0.002	0.003	0.004	0.005	0.006	0.013	0.020	0.030	0.044	0.056	0.072	0.095	0.132	0.156	0.142	0.107	0.073	0.038
S > UFT + GEM	0.256	0.126	0.102	0.107	0.099	0.079	0.059	0.041	0.028	0.025	0.021	0.017	0.014	0.008	0.007	0.004	0.002	0.002	0.001	0.001	0.000	0.000

S, surgery; CT, chemotherapy; RT, radiotherapy; CDDP, cisplatin; GEM, gemcitabine; FFOX, FOLFIRINOX; CAPE, capecitabine; GNABP, gemcitabine + nab-paclitaxel; PEXG, cisplatin + epirubicin + capecitabine + gemcitabine; 5FU, 5-fluorouracil; IFN, interferon; ERLO, erlotinib; UFT, tegafur–uracil; >, followed by.

## Data Availability

The data presented in this study are available upon request from the corresponding author.
